# From Uniaxial Testing of Isolated Layers to a Tri-Layered Arterial Wall: A Novel Constitutive Modelling Framework

**DOI:** 10.1007/s10439-021-02775-2

**Published:** 2021-06-03

**Authors:** Alessandro Giudici, Ashraf W. Khir, Jason M. Szafron, Bart Spronck

**Affiliations:** 1 Biomedical Engineering Theme, Brunel University London, Uxbridge, UK; 2 Department of Biomedical Engineering, Yale University, New Haven, CT USA; 3 Department of Biomedical Engineering, CARIM School for Cardiovascular Diseases, Maastricht University, Universiteitssingel 50, Room 3.359, 6229ER Maastricht, The Netherlands

**Keywords:** Tri-layered arterial wall model, Residual stresses, Layer-specific mechanics, Aorta, Arterial mechanics

## Abstract

Mechanical testing and constitutive modelling of isolated arterial layers yields insight into the individual layers’ mechanical properties, but *per se* fails to recapitulate the *in vivo* loading state, neglecting layer-specific residual stresses. The aim of this study was to develop a testing/modelling framework that integrates layer-specific uniaxial testing data into a three-layered model of the arterial wall, thereby enabling study of layer-specific mechanics under realistic (patho)physiological conditions. Circumferentially and axially oriented strips of pig thoracic aortas (*n* = 10) were tested uniaxially. Individual arterial layers were then isolated from the wall, tested, and their mechanical behaviour modelled using a hyperelastic strain energy function. Subsequently, the three layers were computationally assembled into a single flat-walled sample, deformed into a cylindrical vessel, and subjected to physiological tension-inflation. At the *in vivo* axial stretch of 1.10 ± 0.03, average circumferential wall stress was 75 ± 9 kPa at 100 mmHg, which almost doubled to 138 ± 15 kPa at 160 mmHg. A ~ 200% stiffening of the adventitia over the 60 mmHg pressure increase shifted layer-specific load-bearing from the media (65 ± 10% → 61 ± 14%) to the adventitia (28 ± 9% → 32 ± 14%). Our approach provides valuable insight into the (patho)physiological mechanical roles of individual arterial layers at different loading states, and can be implemented conveniently using simple, inexpensive and widely available uniaxial testing equipment.

## Introduction

The mechanical properties of the arterial wall are highly influenced by the structural arrangements of its constituents. Elastin and collagen are commonly considered the major determinants of the passive mechanical response of arteries, as smooth muscle cells have a relatively low passive stiffness.^5^,^14^,^45^ As reported in several studies, the relative amount, as well as the spatial organisation, of elastin and collagen fibres varies considerably across the arterial wall,^14^,^22^,^25^ conferring different mechanical properties and function to the intimal, medial, and adventitial layers. The intima directly interfaces with the blood flow and has a marginal contribution the overall wall mechanics of young healthy arteries.^5^ The media, characterised by ‘concentric’ elastin lamellae that confer the compliant function to elastic arteries, determines the wall behaviour at physiological pressures.^45^ The adventitia is the outermost, highly collagenous layer that protects arteries from rupture at supraphysiological pressures.^5^,^25^

Arterial mechanical behaviour shows anisotropy, i.e., axial and circumferential mechanical behaviours differ, as well as strong biaxial coupling, where axial loading influences circumferential behaviour and vice versa.^38^ Although biaxial experimental testing, loading the samples in two directions simultaneously, directly yields biaxial mechanical responses, specialised equipment, which is not available in many biomechanics laboratories, is needed. Uniaxial testing, however, is easier to perform, and by combining uniaxial testing data from circumferentially and axially cut arterial strips, biaxial behaviour can be investigated without assessment of the direct coupling. This method has been used in several locations along the arterial tree and in different species, including human^43^ and pig^29^ thoracic aortas, aneurysmal human ascending aortas,^33^ and human coronary arteries.^19^ Furthermore, testing of isolated arterial layers allows for investigating the impact of different layers’ microstructure on arterial mechanics. By fitting structurally motivated hyperelastic strain energy functions (SEFs, i.e., constitutive models whose parameters reflect the mechanical behaviour of the arterial wall under conditions of interest) to the experimental data, such studies^19^,^29^,^33^,^43^ have shown how the different microstructural features of the three layers strongly affect their mechanical properties, including the degree of anisotropy and the recruitment of collagen fibres. Layer-specific mechanical testing and modelling of small arteries (e.g., coronary arteries) has also been successfully performed using more complex pseudo-physiological loading conditions involving inflation, axial extension and twist of tubular samples.^17^,^18^,^41^

While characterising isolated layers provides some insight, understanding the individual layers’ respective roles in the overall mechanical behaviour of the arterial wall is required to further our understanding of (patho)physiology. The standard approach to this problem consists of formulating SEFs that account for the contribution of wall constituents (e.g., collagen and elastin) in each modelled arterial layer and fitting to the experimental whole-wall mechanical behaviour.^30^,^42^ However, the number of constitutive parameters increases with the complexity of the model, increasing the risk of overfitting. More fundamentally, a stress split between the individual layers cannot be inferred from whole-wall mechanical testing without additional structural information and assumptions.^2^

Histological images of the wall cross-section are often used to infer structural features of the wall constituents and constrain model parameters. For example, Polzer *et al.*^30^ implemented a two-layered model of the arterial wall with an isotropic neo-Hookean SEF in the media reinforced by medial and adventitial collagen fibres with anisotropic SEFs. In their work, collagen fibre orientation was inferred from histological images, and probability functions describing collagen recruitment were postulated from the literature and from a qualitative observation of collagen waviness across the wall thickness. Fata *et al.*^11^,^12^ chose anisotropic SEFs for modelling both collagen and elastin and used multi-photon fluorescence of the ovine pulmonary artery wall tissue subjected to biaxial testing to determine collagen and elastin orientation distributions. Moreover, the waviness of the adventitial collagen was used to define a probabilistic recruitment function describing its delayed response. Wang and colleagues^6^,^42^ extended this approach to multi-layer modelling, including contribution of medial elastin and medial and adventitial collagen fibres. Further, Rego *et al*.^32^,^39^ combined layer-specific biaxial mechanical testing and tissue imaging, allowing the quantification of the transmural variation of constituent volumetric fractions and of the fibre orientation distribution function, to develop a multi-layered model of the aortic valve leaflet. Another class of models, exemplified by Witzenburg *et al*.^44^ and Mahutga and Barocas,^28^ examine aortic biomechanical behaviour using a representative volume element (RVE) approach, where the wall includes a series of lamellar units akin to the elastic lamellae of the media. Each RVE includes a discrete network model representing fibres of collagen, elastin, and interlamellar connections. While these studies represent comprehensive approaches to multi-layer modelling, the need for detailed microstructural information from complex and expensive imaging techniques (e.g., multiphoton microscopy) makes wide adoption of these methods infeasible and limits the scope of available data. Further, the opacity of the arterial wall tissue limits the penetration depth of most microscopy techniques, so that, in human-like arteries, only superficial regions (~ 100 $\mu$m) of the samples can be imaged.^6^,^25^

The aim of this study was to develop a testing/modelling framework that integrates layer-specific uniaxial testing data into a three-layered model of the arterial wall, thereby enabling study of layer-specific mechanics under realistic (patho)physiological conditions using simple, widely available and inexpensive experimental techniques. The wall was assumed to comprise three thin-walled concentric layers, each modelled as an isotropic matrix reinforced by two symmetrically oriented fibre families.^43^ Layer-specific SEF parameters were fit using uniaxial testing data obtained in the circumferential and axial directions on isolated layers; layers were then assembled computationally so that the sum of their contributions matched the uniaxial response of the intact wall in both directions. Finally, the resulting tri-layered arterial wall was ‘closed’ into a cylindrical vessel and loaded to physiological conditions, enabling study of the individual layers’ contributions to *in vivo* arterial mechanics.

## Materials and Methods

### Theoretical Background

#### Tri-Layered Wall Model

**Figure 1:**
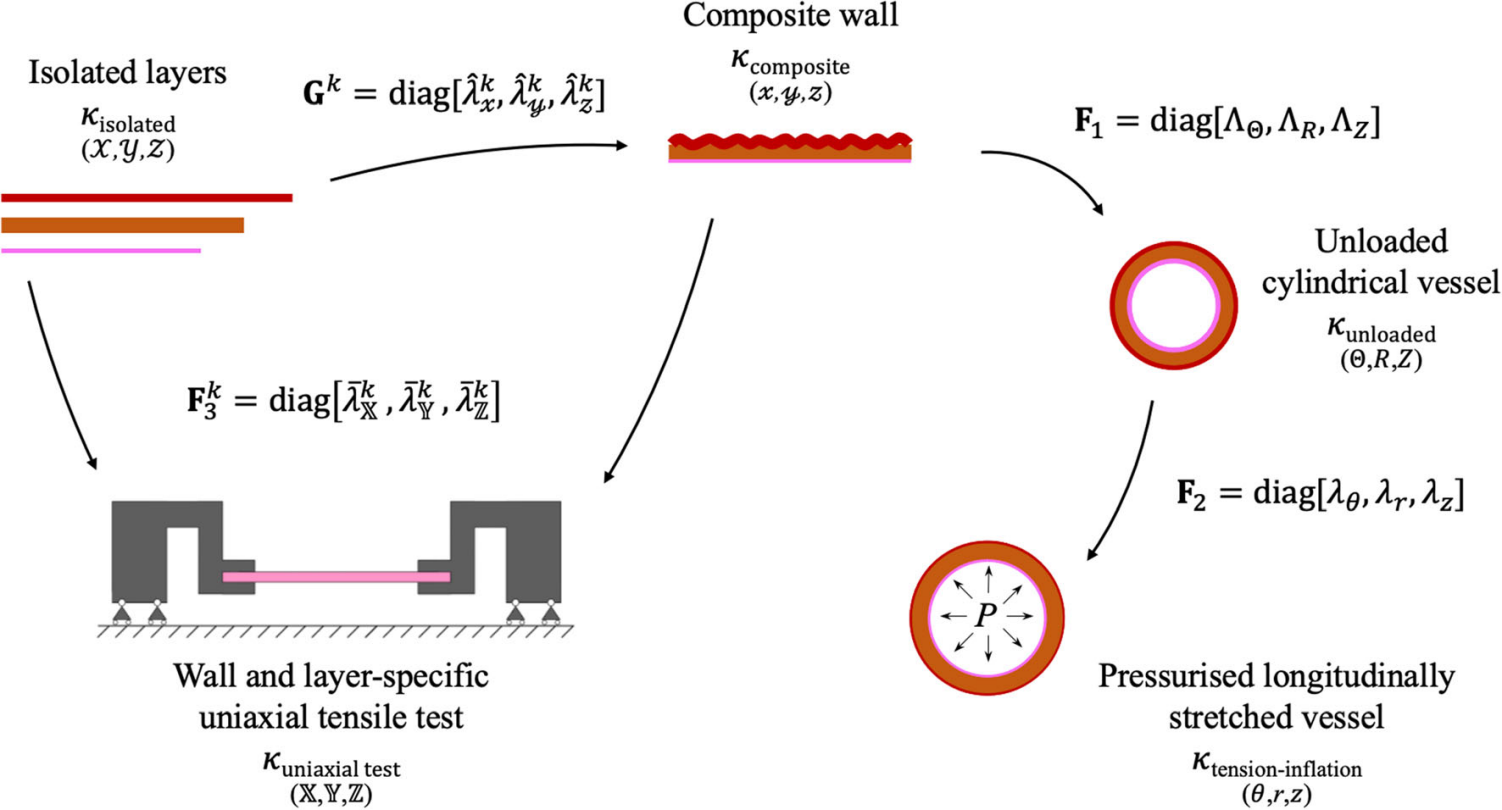
Schematic representation of the mapping flow linking the different configurations analysed in this study: (1) isolated layers ($\kappa_{\text{isolated}}$), (2) composite wall ($\kappa_{\text{composite}}$), (3) unloaded cylindrical vessel ($\kappa_{\text{unloaded}}$), and (4) pressurised axially stretched cylindrical vessel ($\kappa_{\text{tension} - \text{inflation}}$). Coordinates used in the respective configurations are given between parentheses. Superscript $k \in \left\{{\text{i,\;m,\;a,\;wall}} \right\}$ indicates the intimal (i), medial ($\text {m}$), or adventitial ($\text {a}$) layer, or the whole wall (only for **F**^**k**^_**3**_) respectively.

The wall was comprised of three adequately spaced membranes corresponding to the three arterial layers. Composition from the isolated layer to a pressurised cylindrical vessel requires three mapping steps as represented in Fig. 1.

First, considering a flat rectangular slab of excised arterial wall tissue, each layer can be deformed in both circumferential and axial directions. Therefore, when isolated from the wall, the layer releases such prestresses, leading to a layer-specific deformation gradient ${\mathbf{G}}^{k}$ (where $k \in \left\{{{\text{i}},{\text{m}},{\text{a}}} \right\}$, i = intima, m = media, and a = adventitia) mapping the deformation from $\kappa_{\text{isolated}}$ in Cartesian coordinates $(\mathcal{X}, \mathcal{Y}, \mathcal{Z} )$ to $\kappa_{\text{composite}}$ in $(\fancyscript{x}, \fancyscript{y}, \fancyscript{z} )$. We assumed that the layer separation induces negligible shear deformations and, hence, the only non-zero components of ${\mathbf{G}}^{k}$ are those in the three principal directions.

For each layer *k*,

1 $$ {\mathbf{G}}^{k} = {\text{diag}}\left[{\begin{array}{*{20}c} {\frac{{l_{\fancyscript{x}}}}{{L_{{\mathcal{X}}}^{k}}},} & {\frac{{L_{{\mathcal{X}}}^{k} L_{{\mathcal{Z}}}^{k}}}{{l_{\fancyscript{x}} l_{\fancyscript{z}}}},} & {\frac{{l_{\fancyscript{z}}}}{{L_{{\mathcal{Z}}}^{k}}}} \\ \end{array}} \right] = {\text{diag}}\left[{\begin{array}{*{20}c} {\hat{\lambda}_{\fancyscript{x}}^{k},} & {\frac{1}{{\hat{\lambda}_{\fancyscript{x}}^{k} \hat{\lambda}_{\fancyscript{z}}^{k}}},} & {\hat{\lambda}_{\fancyscript{z}}^{k}} \\ \end{array}} \right], $$

where $L_{{\mathcal{X}}}^{k}$ and $L_{{\mathcal{Z}}}^{k}$ are the circumferential and axial lengths of isolated layer *k*, $l_{\fancyscript{x}}$ and $l_{\fancyscript{z}}$ are the circumferential and axial length of the composite wall, $\hat{\lambda}_{\fancyscript{x}}^{k} = l_{\fancyscript{x}}/L_{{\mathcal{X}}}^{k}$ and $\hat{\lambda}_{\fancyscript{z}}^{k} = l_{\fancyscript{z}}/L_{{\mathcal{Z}}}^{k}$ are the stretches in the circumferential and axial direction, and the radial component is determined from incompressibility. Note, ${\mathbf{G}}^{k}$ is homogeneous through the thickness of each layer.

The next deformation gradient ${\mathbf{F}}_{1}$ maps the flat composite wall, $\kappa_{\text{composite}}$, into a (closed) cylindrical vessel, $\kappa_{\text{unloaded}}$ in cylindrical coordinates (${\Theta}$, *R*, *Z*). It is assumed that the entire wall is subjected to the same axial stretch, ${\Lambda}_{Z}$, relative to $\kappa_{\text{composite}}$. The circumferential stretch can be determined by enforcing conservation of volume. Expressing *R* as a function of the flat wall geometry and ${\Lambda}_{Z}$

2 $$ R=\sqrt{R_{\text{internal}}^2+\frac{{l_{\fancyscript{x}}}{\fancyscript{y}}}{\pi\Lambda_Z}}~, $$

the circumferential stretch (${\Lambda}_{\Theta}$) becomes

3 $$ \Lambda_\Theta=\frac{2\pi R}{l_{\fancyscript{x}}}=\sqrt{\frac{4\pi^2R_{\text{internal}}^2}{l_{\fancyscript{x}}^2}+\frac{4\pi{\fancyscript{y}}}{l_{\fancyscript{x}}\Lambda_Z}}~.$$

The deformation gradient ${\mathbf{F}}_{1}$ is therefore defined as

4 $$ {\mathbf{F}}_{1} = {\text{diag}}\left[{\begin{array}{*{20}c} {{\Lambda}_{\Theta},} & {\frac{1}{{{\Lambda}_{\Theta} {\Lambda}_{Z}}},} & {{\Lambda}_{Z}} \\ \end{array}} \right]. $$

An additional deformation gradient ${\mathbf{F}}_{2}$ maps the tension-inflation of the vessel to its *in vivo* configuration ($\kappa_{{{\text{tension}} - {\text{inflation}}}}$). Again, the axial deformation for this motion, ${\lambda}_{z}$, is assumed constant throughout the wall, and the circumferential deformation can be inferred from conservation of volume:

5 $$ {\lambda}_{\theta} = \frac{r}{R} = \sqrt {\left({\frac{{r_{\text{internal}}}}{R}} \right)^{2} + \frac{{R^{2} - R_{\text{internal}}^{2}}}{{R^{2} \lambda_{z}}}}\,. $$

Therefore, ${\mathbf{F}}_{2}$ can be formulated as

6 $$ {\mathbf{F}}_{2} = {\text{diag}}\left[{\begin{array}{*{20}c} {\lambda_{\theta},} & {\frac{1}{{\lambda_{\theta} \lambda_{z}}},} & {\lambda_{z}} \\ \end{array}} \right]. $$

The total deformation from $\kappa_{\text{isolated}}$ to $\kappa_{{{\text{tension}} - {\text{inflation}}}}$ for a layer *k* is given by

7 $$ {\mathbf{F}}_{{{\text{total}},k}} = {\mathbf{F}}_{2} {\mathbf{F}}_{1} {\mathbf{G}}^{k} = {\text{diag}}\left[{\begin{array}{*{20}c} {\lambda_{\theta} {\Lambda}_{\Theta} \hat{\lambda}_{\fancyscript{x}}^{k},} & {\frac{1}{{\lambda_{\theta} {\Lambda}_{\Theta} \hat{\lambda}_{\fancyscript{x}}^{k} \lambda_{z} {\Lambda}_{\text{Z}} \hat{\lambda}_{\fancyscript{z}}^{k}}},} & {\lambda_{z} {\Lambda}_{\text{Z}} \hat{\lambda}_{\fancyscript{z}}^{k}} \\ \end{array}} \right]. $$

Finally, we introduce an additional layer and wall-specific deformation gradient mapping the deformation from $\kappa_{\text{isolated}}$ to $\kappa_{\text{uniaxial\, test}}$ and from $\kappa_{\text{composite}}$ to $\kappa_{\text{uniaxial\, test}}$, respectively:

8 $$ {\mathbf{F}}_3^k=\text{diag}\left[\overline{\lambda}_\mathbb{X}^k, \overline{\lambda}_\mathbb{Y}^k, \overline{\lambda}_\mathbb{Z}^k\right] $$

where $k = \left\{{{\text{wall}},{\text{i}},{\text{m}},{\text{a}}} \right\}$ and using Cartesian coordinates $(\mathbb{X}, \mathbb{Y}, \mathbb{Z})$ in $\kappa_{\text{uniaxial\, test}}$.

#### Layer-Specific Constitutive Modelling

The three individual arterial layers were modelled using the Holzapfel–Gasser–Ogden (HGO) two fibre family-SEF.^13^ The HGO-SEF assumes that the passive behaviour of the arterial wall is described well by the sum of two constituents: the first, typically associated with elastin, that exhibits an isotropic behaviour, and a second, collagen, whose behaviour is anisotropic:

9 $$ {{\Psi}} = \mu^{k} \left({I_{1} - 3} \right) + \mathop \sum \limits_{i = 1}^{2} \frac{{c_{1}^{k}}}{{2c_{2}^{k}}}\left({e^{{c_{2}^{k} \left[{\rho^{k} \left({I_{1}} \right) + \left({1 - 3\rho^{k}} \right)I_{4,i} - 1} \right]^{2}}} - 1} \right), $$

where $\mu^{k}$ is an isotropic stiffness-like parameter ($k \in \left\{{{\text{i}},{\text{m}},{\text{a}}} \right\}$, i = intima, m = media, and a = adventitia), $c_{1}^{k}$ is a collagen stiffness-like parameter, $c_{2}^{k}$ is a dimensionless collagen nonlinearity parameter, and $\rho^{k} \in \left[{0,\frac{1}{3}} \right]$ is a fibre dispersion coefficient, with $\rho = 0$ denoting fully aligned and $\rho = \frac{1}{3}$ denoting fully dispersed fibres. $I_{1}$ and $I_{4,i}$ denote the first and fourth invariant of the right Cauchy-Green tensor, respectively, with $i \in \left\{{1,2} \right\}$ indicating the collagen fibre family with principal orientation $\alpha_{1,2}^{k} = \left\{{- \alpha^{k},\alpha^{k}} \right\}$ with respect to the circumferential orientation. Symmetry ($\alpha_{1}^{k} = - \alpha_{2}^{k}$) results in $I_{4,1} = I_{4,2}$, and, hence, Eq. (9) can be simplified to 13:

10 $$ {{\Psi}} = \mu^{k} \left({I_{1} - 3} \right) + \frac{{c_{1}^{k}}}{{c_{2}^{k}}}\left({e^{{c_{2}^{k} \left[{\rho^{k} \left({I_{1}} \right) + \left({1 - 3\rho^{k}} \right)I_{4,i} - 1} \right]^{2}}} - 1} \right). $$

Given Eq. (10), the Cauchy stress tensor can be defined as

11 $$ {\mathbf{t}}^{k} = - p{\mathbf{I}} + 2{\mathbf{F}}\frac{{\partial {{\Psi}}}}{{\partial {\mathbf{C}}}}{\mathbf{F}}^{\text{T}}, $$

where ${\mathbf{I}}$ is the spatial second order identity tensor and *p* is a Lagrange multiplier enforcing incompressibility. Experimental studies have demonstrated that the arterial wall is nearly incompressible.^7^ Therefore, in agreement with previous studies, we extended the incompressibility assumption to the individual layers.^9^,^29^,^43^

While some concerns have been raised regarding the ability of SEFs with discrete fibre orientations to capture the arterial wall mechanical behaviour,^4^ it has been shown previously that inclusion of a fibre dispersion coefficient considerably improves these models.^13^ It is also worth noting that, despite the intimal microstructure being notably different from that of both media and adventitia, the HGO-SEF has been used previously to accurately model the behaviour of all three arterial layers.^29^,^43^

### Experimental Methods

Ten pig plucks (age 6–12 months, sex unknown) were obtained from a local abattoir (Samples for school, UK). Animal organs were delivered frozen and immediately stored at − 20 °C in a laboratory freezer. The pluck was left to thaw at room temperature for approximately 4 h, after which the aorta was carefully dissected from the rest of the organs using a scalpel. At least two circumferentially and two axially oriented ~ 5 mm wide and ~ 25 mm long strips were cut from the upper thoracic aorta of each animal using a scalpel. Width and thickness were measured three times along the strip length using a high precision digital calliper, after which strips were uniaxially tested, pulling in the strip direction. After testing, each strip was carefully peeled into its three anatomical layers (intima, media, and adventitia) using tweezers,^29^,^36^ and each layer was uniaxially tested separately. When peeling resulted in rupture of a layer, when available, said layer was obtained from an adjacent arterial strip.

All uniaxial tests followed the same protocol. Each intact wall or layer strip was mounted on a uniaxial tensile device (MFS Stage with 20 N load cell, Linkam Scientific Ltd., UK), blocking the ends using serrated jaws. Initial inter-jaw distance was 15 mm to ensure an aspect ratio (length/width) above 2 which has been shown to minimise local distortion in uniaxial tests.^10^,^13^ This distance was then adjusted until the sample was flattened (requiring initial force < 0.020 N), and the corresponding inter-jaw distance was set as the unloaded sample length $L_{0}$. Samples were cyclically tested to a peak Cauchy stress value of 250 kPa, and the loading part of the sixth cycle was used for the analysis. Preliminary testing indicated that five preconditioning cycles were sufficient to obtain a repeatable force–elongation curve. The experimental (exp) wall Cauchy stress was calculated as

12 $$ t_{{ii,{ \exp }}}^{k} = \frac{F}{{A_{0}}}\overline{\lambda}_{i}^{k}, $$

where $F$ is the measured force, $A_{0}$ the unloaded cross-sectional area, and $\overline{\lambda}_{i}^{k}$ $i\in\left\{\mathbb{X},\mathbb{Z}\right\}$ and $k \in \left\{{{\text{wall}},{\text{i}},{\text{m}},{\text{a}}} \right\}$) the applied (tensile) stretch (note that, in uniaxial tensile tests, when incompressibility is assumed, the deformed cross-sectional area $A$ equals $A_{0} /\bar{\lambda }_{i}^{k}$).

### Parameter Estimation

**Figure 2:**
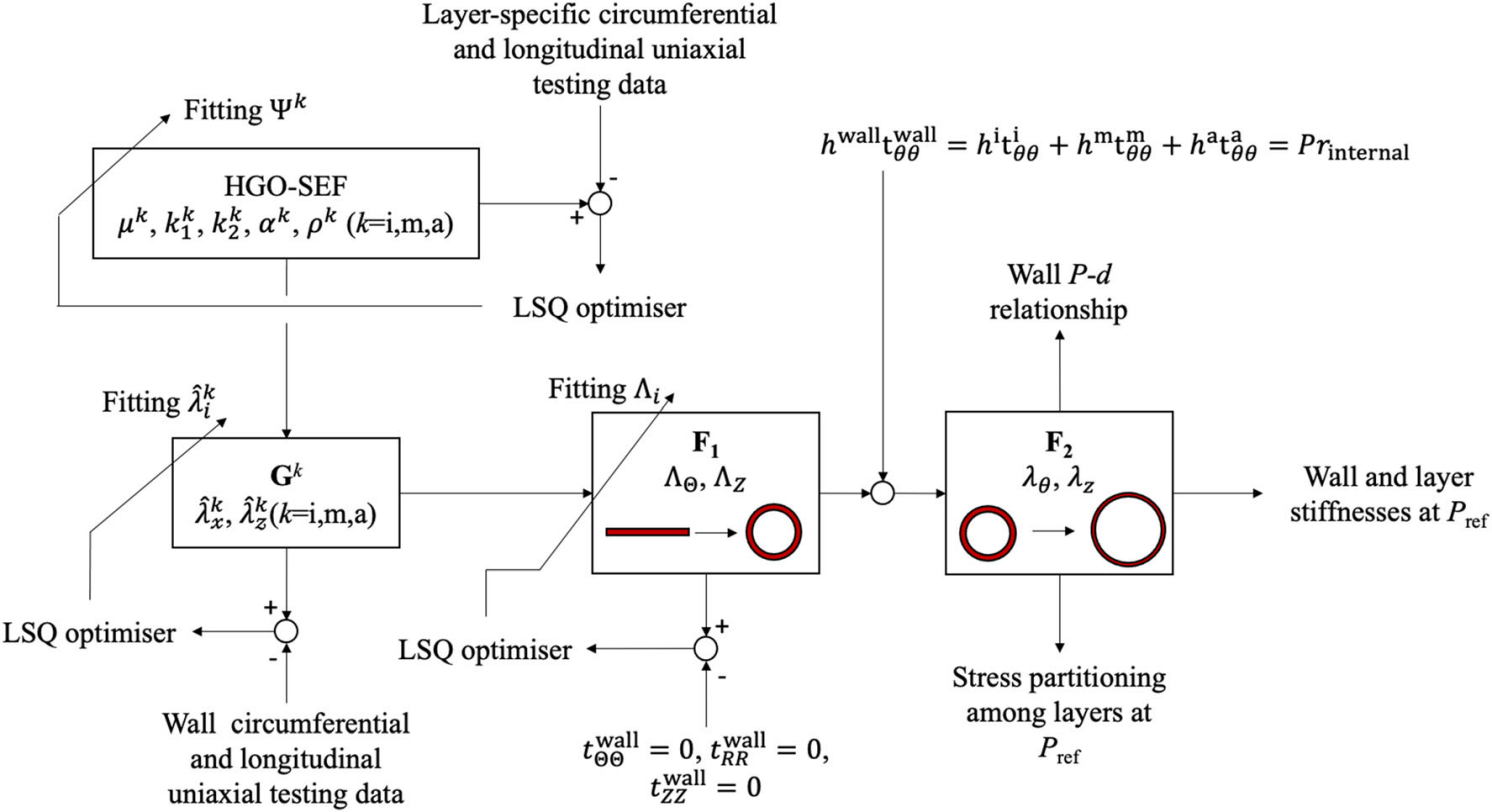
Schematic representation of the workflow proposed in this study. Layer-specific uniaxial testing is used for the optimisation of the layer-specific Holzapfel–Gasser–Ogden Strain Energy Function parameters. The layer-specific models are then combined to match the uniaxial testing of the whole wall, optimising the deformation gradients $\text{G}^{k}$. The deformation gradient $\text{F}_{1}$ describes the closure of the flat wall into a cylindrical segment, satisfying zero average stress in the three principal directions. The deformation gradient $\text{F}_{2}$ provides the tension-inflation of the cylindrical vessel.

Figure 2 presents methods used for fitting the model parameters. First, the layer-specific constitutive parameters in Eq. (10) were fitted to minimise the error between the measured and estimated stress in the loading direction for both the $\mathbb{X}$ and $\mathbb{Z}$ uniaxial tests, simultaneously. To give the same weight to the stress–stretch relationships resulting from the uniaxial test in the $\mathbb{X}$ and $\mathbb{Z}$ directions, each stress–stretch relationship was resampled at 50 equally-spaced increments between $\bar{\lambda}_{i}^{k} = 1$ and the maximum stretch, leading to the following cost function:

13 $$J=\sum_{m=1}^{50}\left(t_{\mathbb{XX},\text{exp}}^k(m)-t_\mathbb{XX}^k(m)\right)^2+\sum_{n=1}^{50}\left(t_{\mathbb{ZZ},\text{exp}}^k(n)-t_\mathbb{ZZ}^k(n)\right)^2.$$

In a uniaxial tensile test, the only non-zero component of the Cauchy stress tensor is the component corresponding to the loading direction $\bar{\lambda}_{i}^{k}$. $\bar{\lambda}_{j}^{k}$ is determined by imposing the off-axis stress $t_{jj}^{k} = 0$ and enforcing incompressibility. The fourth-invariant in Eq. (10) was then $I_{4,i}=\left(\overline{\lambda}_\mathbb{X}^k\right)^2 \cos^2(\alpha_i)+\left(\overline{\lambda}_\mathbb{Z}^k\right)^2 \sin^2(\alpha_i)$.

The deformation tensors ${\mathbf{G}}^{k}$, $k \in \left\{{{\text{i}},{\text{m}},{\text{a}}} \right\}$ require the estimation of six stretches $\hat{\lambda}_{i}^{k}$ (three layers times two stretches) defining the axial and circumferential prestretches that each layer is subjected to when part of the wall. $\hat{\lambda}_{i}^{k}$ was estimated by minimising the error between the experimental and modelled wall stresses in the circumferential and axial directions, simultaneously (i.e., minimising the cost function *J*). The average Cauchy stress of the composite wall ($\kappa_{\text{composite}}$) was calculated as

14 $$ {\mathbf{t}}^{\text{wall}} = \frac{{h^{\text{i}} {\mathbf{t}}^{\text{i}} + h^{\text{m}} {\mathbf{t}}^{\text{m}} + h^{\text{a}} {\mathbf{t}}^{\text{a}}}}{{h^{\text{wall}}}} $$

with layer thicknesses $h^{k}$ determined using the layer thickness in $\kappa_{\text{isolated}}$ and enforcing incompressibility. Additionally, the search for $\hat{\lambda}_{i}^{k}$ was constrained to ranges measured experimentally on a separate cohort of arterial samples. As described in the experimental methods, mounting the samples on the uniaxial tensiometer requires setting its unloaded length. Since the components of ${\mathbf{G}}^{k}$ were fitted on the basis of the mechanical data, the modelled $\hat{\lambda}_{i}^{k}$ refers to the ratio between the lengths of the wall and the layer at the beginning of the uniaxial test and might not correspond exactly to that measured experimentally due to the possible under/over-estimation of the sample’s unloaded length. For this reason, the constraints were set to mean ± 3 standard deviations of the experimental values (Table 1).

**Table 1:** Experimental and modelled layer pre-stretches.

	Intima		Media		Adventitia	
	$\hat{\lambda}_{\fancyscript{x}}^{\text{i}}$	$\hat{\lambda}_{\fancyscript{z}}^{\text{i}}$	$\hat{\lambda}_{\fancyscript{x}}^{\text{m}}$	$\hat{\lambda}_{\fancyscript{z}}^{\text{m}}$	$\hat{\lambda}_{\fancyscript{x}}^{\text{a}}$	$\hat{\lambda}_{\fancyscript{z}}^{\text{a}}$
Experimental	$1.00 \pm 0.01$	$1.00 \pm 0.01$	$1.00 \pm 0.01$	$0.98 \pm 0.02$	$0.93 \pm 0.02$	$1.01 \pm 0.01$
Modelled	$1.01 \pm 0.02$	$0.99 \pm 0.04$	$1.02 \pm 0.02$	$0.99 \pm 0.02$	$0.95 \pm 0.04$	$1.04 \pm 0.02$

	${\Lambda}_{\Theta}^{\text{i}}$	${\Lambda}_{Z}^{\text{i}}$	${\Lambda}_{\Theta}^{\text{m}}$	${\Lambda}_{Z}^{\text{m}}$	${\Lambda}_{\Theta}^{\text{a}}$	${\Lambda}_{Z}^{\text{a}}$
Modelled	$0.89 \pm 0.01$	$1.01 \pm 0.01$	$0.99 \pm 0.00$	$1.01 \pm 0.01$	$1.09 \pm 0.01$	$1.01 \pm 0.01$

$\hat{\lambda }_{i}^{k}$ indicates the components ($i \in \left\{{\fancyscript{x},\fancyscript{z}} \right\}$) of the layer-specific deformation ${\mathbf{G}}^{k}$ (where $k \in \left\{{{\text{i}},{\text{m}},{\text{a}}} \right\}$, ${\text{i}}$ = intima, ${\text{m}}$ = media, and ${\text{a}}$ = adventitia) mapping the deformation from $\kappa_{\text{isolated}}$ to $\kappa_{\text{composite}}$. ${\Lambda}_{i}^{k}$ indicates the components of the deformation gradient ${\mathbf{F}}_{1}$ mapping the deformation from $\kappa_{\text{composite}}$ to $\kappa_{{{\text{tension}} - {\text{inflation}}}}$. Note that the $\fancyscript{x}$- and $\fancyscript{z}$-direction correspond with the circumferential and axial directions of the intact vessel, respectively. Data are presented as mean ± standard deviation

${\mathbf{F}}_{1}$ maps the deformation of a generic point in the κ_composite_ configuration to the κ_unloaded_ configuration. It can be shown that the $\fancyscript{y}$ coordinate providing the layer-specific ${\Lambda}_{\Theta}$ corresponding to the mid-wall point depends on the deformed configuration itself, and, therefore, ${\Lambda}_{\Theta}$ was estimated iteratively by imposing zero average stress in all three principal directions ($t_{\Theta \Theta}^{\text{wall}} = 0$, $t_{RR}^{\text{wall}} = 0$, $t_{ZZ}^{\text{wall}} = 0$) and satisfying the geometrical constraints determined by the interaction between layers.

Previous studies have shown that the *in vivo* $\lambda_{z}$ is the axial stretch that results in an approximately constant axial force in the physiological range of pressures.^40^ In practice, $\lambda_{z}$ can be estimated as the cross-over point between reduced axial force-axial stretch relationships at different levels of distending pressure, where the reduced axial force is calculated as

15 $$ F_{z} = \pi t_{zz}^{\text{wall}} \left({r_{\text{external}}^{2} - r_{\text{internal}}^{2}} \right) - \pi r_{\text{internal}}^{2} P, $$

with $t_{zz}^{\text{wall}}$ the axial wall stress and $P$ the luminal pressure. $\lambda_{z}$ was estimated as the average between the stretches at cross-over points between relationships at $P$ = 60, 100, and 140 mmHg. $r_{\text{internal}}$ was estimated iteratively so that the desired $P$ is achieved, with *P* calculated using

16 $$ P = t_{\theta \theta} \frac{{r_{\text{external}} - r_{\text{internal}}}}{{r_{\text{internal}}}}. $$

Two reference pressure levels were taken into consideration: $P_{\text{ref}}$ = 100 mmHg representing a normotensive mean arterial pressure, and $P_{\text{ref}}$ = 160 mmHg representing a hypertensive systolic blood pressure. At these pressure levels, layer stresses in the circumferential and axial directions were calculated using Eq. (11), while wall stresses were calculated from the layers’ stresses using Eq. (14). The circumferential material stiffness was calculated according to two different formulations: first, ${\mathcal{K}}_{\theta \theta \theta \theta}$ was calculated as the tangent elastic modulus in the circumferential stress–circumferential strain relationship,^24^ and, second, ${\mathcal{C}}_{\theta \theta \theta \theta}$ was calculated according to the small-on-large formulation^1^:

17 $$ {\mathcal{K}}_{{\theta \theta \theta \theta }} = \left. {\frac{{\partial t_{{\theta \theta }} }}{{\partial \varepsilon _{{\theta \theta }} ~}}} \right|_{{P = P_{{{\text{ref}}}} }} ,{\text{~and}} $$

18 $$ {\mathcal{C}}_{\theta \theta \theta \theta} = 2\left({t_{\theta \theta} + p} \right) + 4\lambda_{\theta}^{4} \frac{{\partial^{2} {{\Psi}}}}{{\partial \left({\lambda_{\theta}^{2}} \right)^{2}}}, $$

where $$ \varepsilon _{{\theta \theta }} = \lambda _{\theta } - 1 $$.

The structural stiffness was calculated as the product between the layer/wall material stiffness and its respective loaded thickness (${\mathcal{K}}_{\theta \theta \theta \theta}^{k} h^{k}$ and ${\mathcal{C}}_{\theta \theta \theta \theta}^{k} h^{k}$ for stiffnesses defined in Eqs. 17 and 18, respectively, and with $k = \left\{{{\text{wall}},{\text{i}},{\text{m}},{\text{a}}} \right\}$). Additionally, the contribution of each layer to the load bearing was calculated as the ratio between the force per unit length of the layer and the wall:

19 $$ {\text{Load bearing}} \% = \frac{{t_{\theta \theta}^{k} h^{k}}}{{t_{\theta \theta}^{\text{wall}} h^{\text{wall}}}} \cdot 100\% $$

To illustrate the role of the layers’ prestretches (i.e., composition of ${\mathbf{F}}_{1}$ and ${\mathbf{G}}^{k}$), we also evaluated the case with both ${\mathbf{F}}_{1}$ and ${\mathbf{G}}^{k}$ equal to the spatial second order identity tensor ${\mathbf{I}}$.

Finally, we evaluated the layer-specific stored elastic energy change ($\Delta {{\Psi}}$) over a simulated normotensive (120/80 mmHg systolic/diastolic pressure) and hypertensive (160/100 mmHg) cardiac cycle. Further, the elastic energy was also calculated per unit length by multiplying with the dissected layer cross-sectional area.

### Statistical Analysis

On each aorta, tensile tests were conducted in duplicate on two adjacent circumferential and two adjacent axial strips, as were the layer tests. For each artery, the layer-specific constitutive and tri-layered modelling was then conducted in pairs (i.e., circumferential strip 1 with axial strip 1 and circumferential strip 2 with axial strip 2). For each output variable, the average was considered as the representative value for that artery and used in further group statistical analysis.

Results are presented as mean ± standard deviation of the 10 arteries. Differences in output variables (constitutive parameters, stresses, stiffnesses) among arterial layers were first evaluated using a permissive repeated-measures analysis of variance (ANOVA) followed by paired student’s t-tests for the pairwise comparisons. *p *< 0.05 was taken as statistically significant. In null hypothesis significance testing, *p* indicates the probability of incorrectly rejecting the null hypothesis.

## Results

### Layer-Specific Modelling

The average unloaded radius and wall thickness of the pig upper thoracic aorta were 8.66 ± 0.74 mm and 2.23 ± 0.21 mm, respectively. The layers’ thicknesses were 0.33 ± 0.07 mm for the intima, 1.35 ± 0.19 mm for the media, and 0.61 ± 0.06 mm for the adventitia, corresponding to 14 ± 3, 59 ± 4, and 27 ± 2% of the wall thickness, respectively.

**Figure 3:**
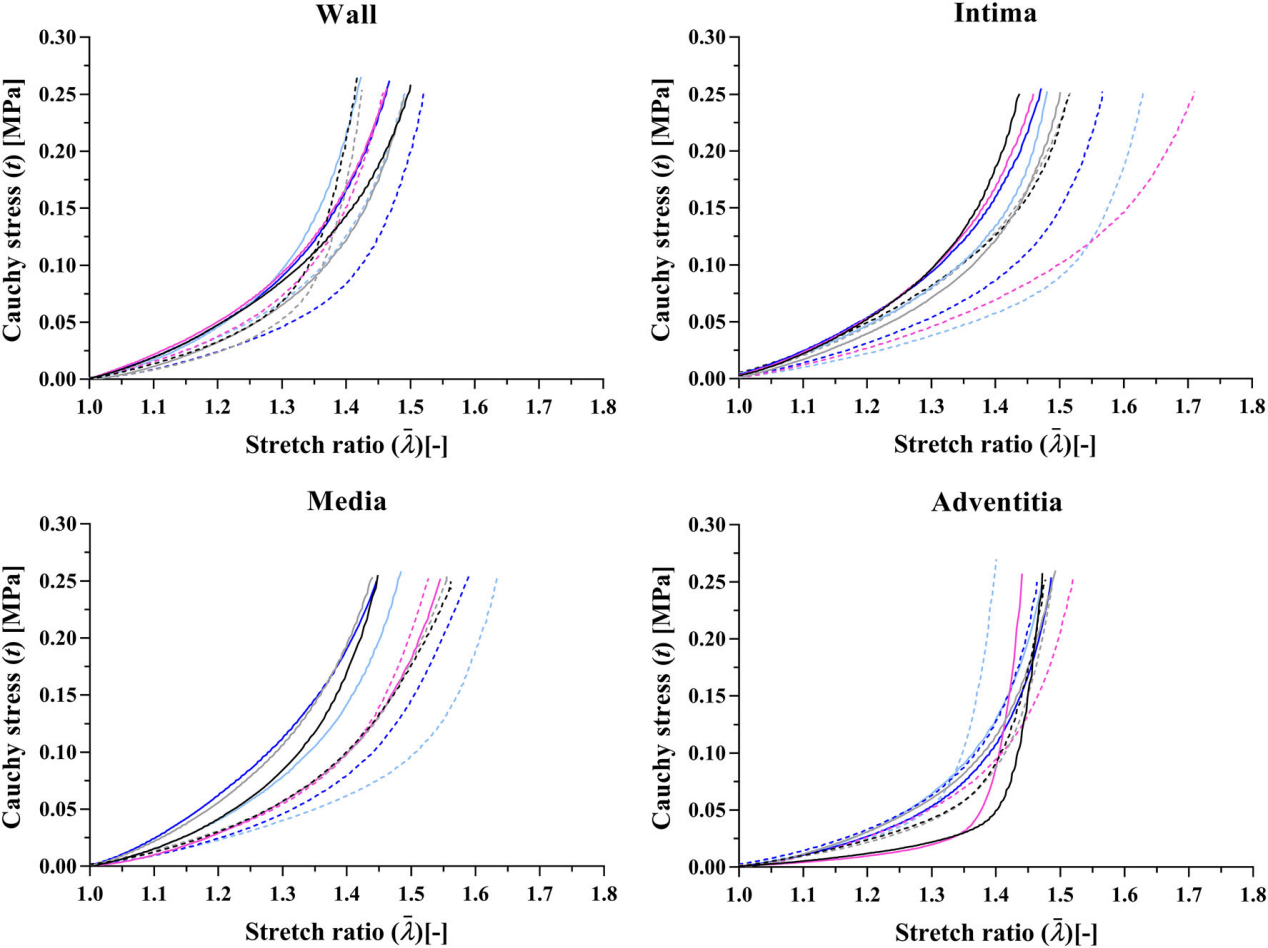
Representative wall and individual-layer stress–stretch relationships of five of the pig upper thoracic aortas included in this study. Solid and dashed lines indicate uniaxial tests in the circumferential and axial directions, respectively.

Figure 3 shows example stress–stretch relationships for the intact wall and isolated layers, and Table 2 presents the HGO-SEF layer-specific parameters fitting. The three layers displayed different mechanical behaviours; the media showed the highest level of anisotropy ($\alpha \approx 35^\circ)$, while the adventitial response was almost isotropic ($\alpha \approx 45^\circ$). Also, the adventitial collagen parameters $c_{1}$ and $c_{2}$ were smaller and larger, respectively, than those of the other layers, suggesting a delayed and more abrupt recruitment of fibres in the outermost layer (Fig. 3).

**Table 2:** Layer-specific Holzapfel–Gasser–Ogden model parameters of the pig upper thoracic aortas included in this study.

	Sample #	$\varvec{\mu}^{\varvec{k}}$ [kPa]	$\varvec{c}_{1}^{\varvec{k}}$[kPa]	$\varvec{c}_{2}^{\varvec{k}}$[-]	$\varvec{\alpha}^{\varvec{k}}$ [˚]	$\varvec{\rho}^{\varvec{k}}$ [-]	*R*^2^
Intima	I	31.1	119.2	17.5	40.8	0.24	0.99
	II	22.3	158.1	4.7	40.2	0.21	0.98
	III	22.8	121.0	4.3	33.0	0.23	1.00
	IV	26.9	78.9	15.4	44.7	0.27	1.00
	V	20.9	125.3	11.0	30.4	0.23	1.00
	VI	34.2	81.9	19.4	43.3	0.26	0.99
	VII	25.5	86.3	6.7	37.1	0.26	0.99
	VIII	18.9	55.8	8.7	45.7	0.28	0.98
	IX	22.2	157.0	10.2	42.7	0.22	1.00
	X	12.2	189.1	4.8	41.7	0.20	0.99
	Mean ± SD	23.7 ± 5.9	117.3 ± 39.9	10.3 ± 5.2	39.9 ± 4.7	0.24 ± 0.03	0.99 ± 0.01
	Av. response	23.5	124.7	11.0	39.5	0.24	1.00
Media	I	19.8	200.4	7.7	40.3	0.21	0.99
	II	21.8	103.4	6.5	27.0	0.26	1.00
	III	21.5	129.0	6.1	20.4	0.26	1.00
	IV	19.0	163.6	3.6	33.0	0.23	1.00
	V	36.0	108.0	18.2	30.1	0.22	1.00
	VI	14.5	199.9	2.0	39.4	0.20	0.99
	VII	14.6	120.0	5.0	37.5	0.22	0.99
	VIII	11.5	135.6	1.3	36.0	0.23	0.99
	IX	20.1	176.6	2.8	37.4	0.21	1.00
	X	24.7	126.2	5.3	34.8	0.26	1.00
	Mean ± SD	20.3 ± 6.5	146.3 ± 34.4	5.8 ± 4.5	33.6 ± 5.9	0.23 ± 0.02	1.00 ± 0.00
	Av. response	22.3	134.8	9.5	33.4	0.24	1.00
Adventitia	I	26.0	156.4	26.2	45.7	0.21	0.99
	II	15.6	95.3	28.2	39.5	0.23	0.98
	III	17.3	51.5	48.3	46.1	0.22	0.96
	IV	27.3	84.4	28.6	47.5	0.23	1.00
	V	9.6	25.1	103.7	40.4	0.20	0.98
	VI	24.0	45.8	41.3	51.1	0.24	0.99
	VII	10.6	92.1	17.5	39.2	0.23	0.99
	VIII	18.3	24.8	53.1	44.9	0.26	1.00
	IX	18.1	80.3	21.0	45.4	0.27	1.00
	X	18.4	82.6	30.9	45.4	0.24	0.96
	Mean ± SD	18.5 ± 5.6	73.8 ± 37.3	39.9 ± 23.9	44.5 ± 3.6	0.23 ± 0.02	0.99 ± 0.01
	Av. response	21.2	25.7	67.3	42.9	0.18	1.00

Mean ± SD (standard deviation) denotes the statistical mean and standard deviation of the parameter values of all ten samples. Av. (average) response denotes the (single) parameter value fitted to the average mechanical response. The average response was determined by averaging the modelled behaviour of samples I–X in both circumferential and axial directions up to a Cauchy stress of 250 kPa

### Wall Modelling

Table 1 presents the modelled circumferential and axial deformations that layers are subjected to when assembled in a flat arterial sample (${\mathbf{G}}^{k}$) and during closure into a three-layered cylindrical structure (${\mathbf{F}}_{1}$). $\hat{\lambda}_{\fancyscript{x}}^{k}$ and $\hat{\lambda}_{\fancyscript{z}}^{k}$ were close to 1 for both the intima and media, but the adventitia was subjected to a 0.95 compressive stretch in the circumferential direction and a 1.04 tensile stretch in the axial direction. The average *R*^2^ of the three-layered flat wall model was 0.99 ± 0.01. As expected, ${\mathbf{F}}_{1}$ led to compression in the intima and tension in the adventitia (Table 1).

**Figure 4:**
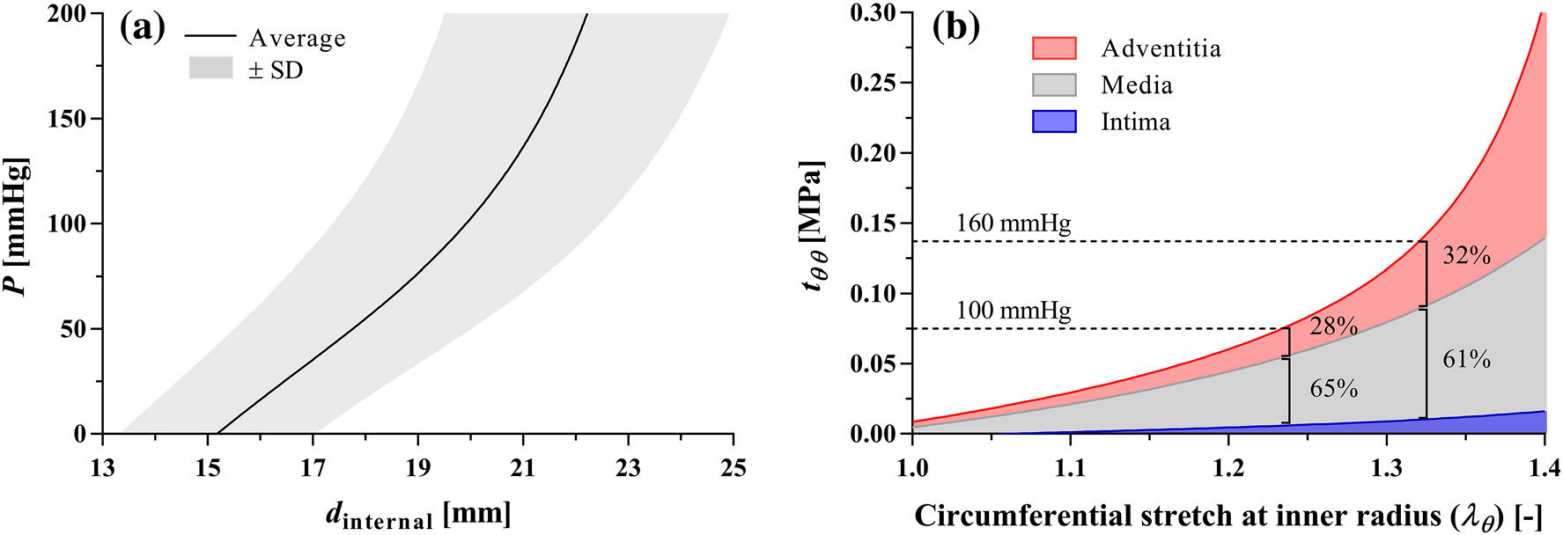
Average pressure-diameter relationship (a) and Cauchy stress–stretch relationship with load partitioning between layers (b) of the 10 aortas tested in this study. Circumferential stretch at inner radius was computed as $\lambda_{\theta} = r_{\text{internal}}/R_{\text{internal}}$. The intimal line was obtained using Eq. (14) with $\varvec{t}^{\text{m}}$ = 0 and $\varvec{t}^{\text{a}}$ = 0, and the media line with $\varvec{t}^{\text{a}}$ = 0. The adventitial line was obtained using the full version of Eq. (14).

**Figure 5:**
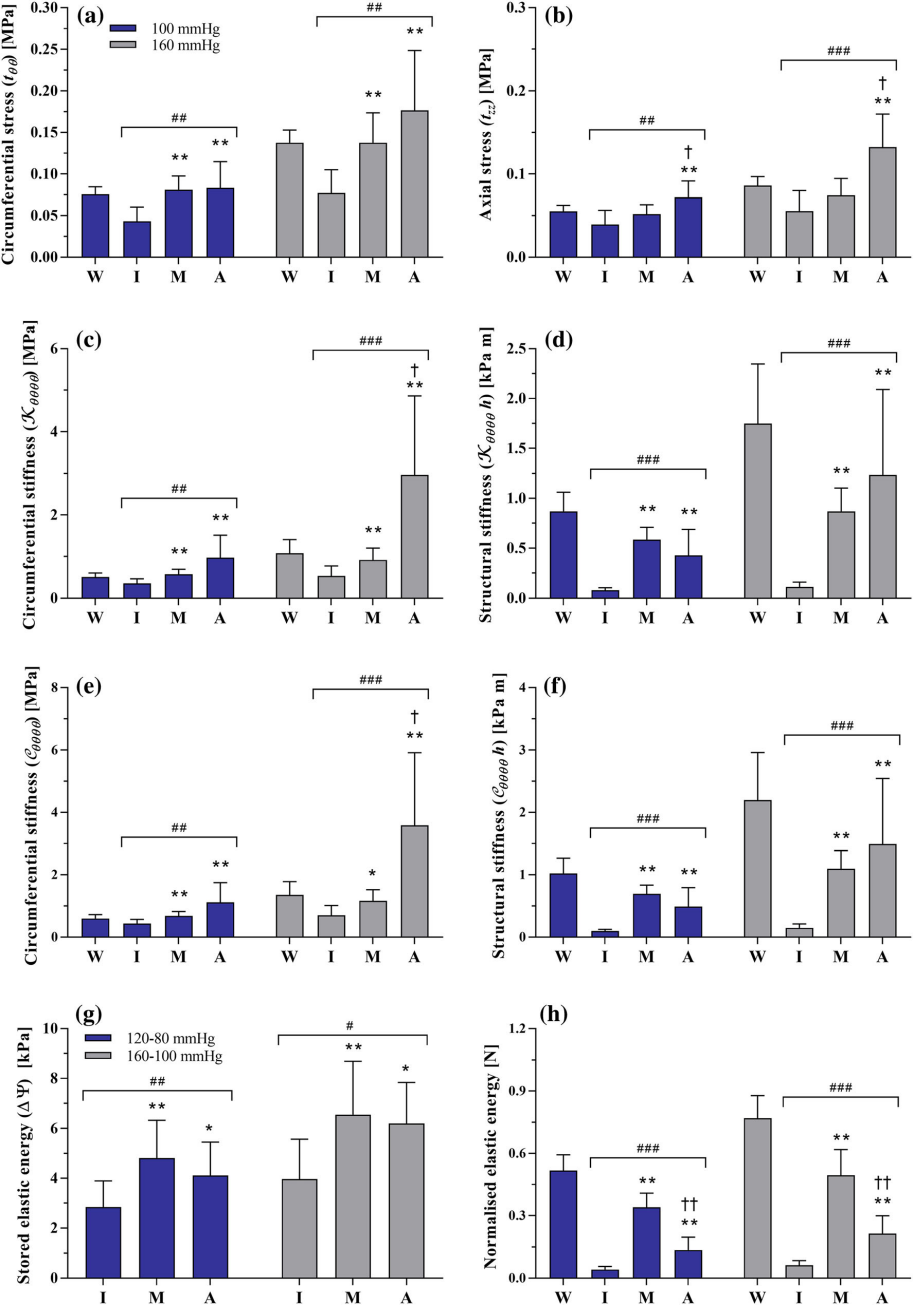
Circumferential (**a**) and axial stress (**b**), circumferential stiffness (**c**, **e**), structural stiffness (**d**, **f**), stored elastic energy (**g**) and stored elastic energy per unit length (**h**) in the pig upper thoracic arch at the reference pressures of 100 and 160 mmHg. W = intact wall, I = intima, M = media, and A = adventitia. Repeated measures ANOVA: ^##^*p *< 0.01 and ^###^*p *< 0.001. Inter-layer pairwise comparisons: *p < 0.05, **p < 0.01 vs. intima, ^†^*p *< 0.05, ^††^*p *< 0.01 vs. media.

Figure 4(a) shows the average pressure-diameter relationship of the composite wall. The *in vivo* axial stretch was 1.10 ± 0.03, while the circumferential stretch at the luminal side of the wall was 1.24 ± 0.04 and 1.33 ± 0.03 at 100 and 160 mmHg, respectively. Figure 5 presents circumferential and axial stresses, and circumferential material and structural stiffness at 100 and 160 mmHg. At 100 mmHg, the average circumferential stress level was very similar in the media and adventitia and comparable to that in the wall (0.075 ± 0.009 MPa). On the contrary, the circumferential stress level in the intima was almost half that of both the media and adventitia (*p *< 0.01). Increasing pressure to 160 mmHg, the wall circumferential stress almost doubled (*p *< 0.001) and became on average higher in the adventitia than that in the media, although the difference was not significant. In the axial direction, the stress was higher in the adventitia than in both the intima and media, independent of the pressure level (*p *< 0.01). At 100 mmHg, the average tangential elastic modulus ${\mathcal{K}}_{\theta \theta \theta \theta}$ for the wall was 0.51 ± 0.09 MPa and increased by 112% at 160 mmHg. Interestingly, this value only increased by 50 and 60% for the intima and media, respectively, over the 60 mmHg pressure increment, while the adventitial value of ${\mathcal{K}}_{\theta \theta \theta \theta}$ tripled (+204%). Similarly, the average linearised stiffness ${\mathcal{C}}_{\theta \theta \theta \theta}$ for the wall was 0.60 ± 0.13 MPa and rose by 127% at 160 mmHg. Changes in ${\mathcal{C}}_{\theta \theta \theta \theta}$ over the 60 mmHg increment were modest for the intima and media (61 and 71%, respectively) and more marked for the adventitia (220%). The stored elastic energy over the cardiac cycle was comparable in the media and adventitia both in the normotensive (4.81 ± 1.52 and 4.11 ± 1.35 kPa, respectively) and hypertensive (6.55 ± 2.14 and 6.20 ± 1.63 kPa, respectively) pressure ranges. When normalised with respect to the cross-sectional area, the media accounted for 67% of the total stored energy in the normotensive (120/80 mmHg) pressure range and slightly lower (65%) in the hypertensive (160/100 mmHg) pressure range. Conversely, the adventitia accounted for 25 and 27% in the normotensive and hypertensive range, respectively, while the intima remained unchanged at 8%.

Figure 4(b) presents the average circumferential Cauchy stress partitioning among layers. At 100 mmHg, 65 ± 10% of the load was borne by the media, 7 ± 3% by the intima, and 28 ± 9% by the adventitia. Increasing pressure to 160 mmHg, the percentage of total load borne by the adventitia increased by 4 percentage points, while decreasing by the same amount in the media. Neglecting the layer prestretches strongly affected stress levels in the three layers, with the highest values in the intima at 0.107 ± 0.025 MPa, 0.084 ± 0.010 MPa in the media, and lowest values in the adventitia at 0.051 ± 0.013 MPa. Consequently, in comparison to the results obtained with the complete model (including prestretches), the intimal load bearing rose to 17 ± 4% while that of the adventitia dropped to 19 ± 4% at 100 mmHg and 21 ± 6% at 160 mmHg.

## Discussion

The composition and structure of the intima, media, and adventitia determine the macroscopic mechanical properties of the arterial wall. While layer-specific constitutive modelling has been performed at different sites along the arterial tree, limited work has used such information for modelling the intact arterial wall. In this study, we proposed a new modelling framework to simulate the response of the arterial wall to inflation and axial extension using the mechanical information gathered from simple uniaxial testing of the three anatomical layers.

The isolation of the arterial wall anatomical layers is a relatively simple process that causes little damage to the isolated structures. Any peeling-induced damage has been shown to be limited to the interconnective tissue between adjacent layers and, hence, will have negligible effects on the layers’ macroscopic behaviour.^19^,^21^,^29^ Values of relative layer thickness found in this study are in agreement with those reported for the porcine proximal thoracic aorta^29^,^35^ and human lower thoracic aorta.^21^ However, previous measurements of the thickness of aortic media and adventitia from histological images suggested that the adventitial layer obtained *via* peeling might be thicker than the anatomical adventitia.^34^ Nevertheless, Peña *et al.*^29^ reported that the percentage of medial lamellar units wrongly included in both the intima and adventitia was minimal and, therefore, such errors in the layer separation are not expected to significantly influence the results.

In agreement with previous results,^43^ the intima, media, and adventitia displayed different levels of anisotropy and rates of collagen recruitment. The media showed early recruitment of collagen fibres and a stiffer response in the circumferential direction, while the adventitia was constitutively almost isotropic and characterised by delayed recruitment of collagen fibres (Fig. 3 and Table 2). This heterogeneity translates into the complex response of the intact wall to uniaxial tensile testing, characterised by a stiffer response in the circumferential direction at low stretch values ($\overline{\lambda}^\text{wall}$ < 1.3) followed by isotropic behaviour for $\overline{\lambda}^\text{wall}$ > 1.3. Interestingly, the fibre orientations in the media and adventitia of the pig upper thoracic aorta reported by Peña *et al.*^29^ are approximately shifted by 10º towards the circumferential direction with respect to those reported here. This could be due to the lower target maximum Cauchy stress used for the constitutive model fitting in their study (~ 0.15-0.17 MPa).

The *in vivo* axial stretch $\lambda_{z}$ is commonly assumed to be near the value of stretch yielding an approximately constant reduced axial force over the physiological range of pressure.^40^ Using this assumption, we obtained $\lambda_{z} = 1.10 \pm 0.03$. Han and Fung^16^ experimentally determined $\lambda_{z}$ in the porcine aorta as a function of the axial position, reporting values ranging ~ 1.1-1.3 in the proximal thoracic aorta, as also confirmed in more recent studies.^29^ At the *in vivo* axial stretch, the infinitesimal stiffness ${\mathcal{K}}$ of the pig aortic wall ranged from ~ 0.5 MPa at 100 mmHg to ~ 1.0 MPa at 160 mmHg, in agreement with values reported previously in tension-inflation studies.^24^,^26^,^27^ Interestingly, the layer-specific analysis showed that pressure-related stiffening was not uniform across the wall thickness, but was maximum in the adventitia (~ 200%) and much smaller in the media (~ 60%), further confirming the adventitia acts as a ‘stress shield’ to prevent rupture at high loads.^3^,^37^ While ${\mathbf{\mathcal{K}}}$ assumes that the non-linear behaviour of the arterial wall can be linearised around the relatively small deformation occurrying within the cardiac cycle, the small-on-large stiffness ${\mathbf{\mathcal{C}}}$ was introduced by Baek *et al.*^1^ to connect the linearised arterial stiffness with complex features of the constitutive relations, such as residual stress, anisotropy, and nonlinear behaviours. This formulation considers the superimposition of a small wall deformation within the cardiac cycle to that necessary to reach the average *in vivo* working point from the stress-free configuration. As expected, values of ${\mathbf{\mathcal{C}}}$ were higher than ${\mathbf{\mathcal{K}}}$, but overall differences between layers were statistically similar in the two formulations. As a result, the stress level was approximately equal in the media and adventitia at the mean physiological pressure of 100 mmHg, in agreement to the theory that arterial remodelling tends to preserve a uniform level of stress across the wall,^23^ while the 60 mmHg increase in pressure resulted in a 4% shift of the circumferential load bearing from the media to the adventitia. These differences indicate that the different layers’ microstructures determine their different functions in arterial mechanics: the media provides compliance to the aortic wall with the elastance necessary to transform the pulsatile flow produced by the heart into a relatively continuous flow by storing energy in systole (65–67% of the total stored energy by the wall). Conversely, the adventitia works as a protective layer, having a marginal contribution at physiological pressure levels, but bearing approximately 40% of the circumferential load at 200 mmHg.

Recently, Diaz *et al.*^9^ found different load distributions for the arterial layers with a similar animal model, though they used a thick-walled model with parameters from a single sample, which highlighted the difficulty in identifying layer-specific opening angles.^20^ Further, uniaxial testing, as well as planar biaxial testing, requires flattening of the samples so that the layer-specific model parameters do not necessarily describe the layer behaviour in its stress-free configuration. Here, we chose a simplified thin-layered modelling framework, where each layer is considered as a membrane and residual deformations are considered as average values across the layer thickness. The layer-specific residual deformation in $\kappa_{\text{unloaded}}$ can be determined by a multiplicative combination of the deformation gradients ${\mathbf{F}}_{1} {\mathbf{G}}^{k}$ (Table 1); where ${\mathbf{G}}^{k}$ describes residual deformations layers are subjected to at the beginning of the wall uniaxial test and ${\mathbf{F}}_{1}$ maps the deformation into a cylindrical vessel. In the circumferential direction, our model predicted a 0.90 compressive stretch ratio for the intima, slight tension for the media, and a 1.04 tensile stretch for the adventitia. The residual deformation gradient found in the circumferential direction is in agreement with previous results indicating that arteries open into an arc shape when cut radially.^21^,^31^ Indeed, Greenwald *et al.*^15^ showed that removal of material from the inside and outside of the arterial wall leads to a decrease and increase, respectively, of the opening angle due to a shift in the equilibrium between tensile and compressive residual stresses. On the contrary, residual deformations in the axial direction were found only in the adventitia (5% tension). While the values of axial prestretch are in line with those reported in Peña *et al.* in the pig upper thoracic aorta,^29^ they also found a similar level of prestretch in the media that we found neither experimentally nor computationally. In agreement with results reported here, neglecting residual defromation when formulating a tri-layered model of the arterial wall results in circumferential stresses monotonically decreasing across the wall thickness and likely leads to overestimation of the contribution of the intima to the overall wall behaviour.^8^

### Limitations

We did not conduct any cross-sectional imaging of the isolated layers. Although the physical separation of arterial layers by peeling is a relatively simple and well-established technique,^29^,^33^,^43^ some sub-optimal layer separation might have occurred. However, layer thicknesses reported here are in agreement with those found in other studies on the pig aorta.^29^,^35^ Therefore, this experimental limitation likely did not affect the validity of our results.

The constitutive parameters of the isolated layers were estimated by fitting simultaneously the stress–stretch relationships resulting from the uniaxial tensile testing in the circumferential and axial directions as done previously.^19^,^43^ The deformation in the other principal direction (i.e., axial and circumferential, respectively) was not measured but determined by enforcing incompressibility and zero traction in all principal directions except that of the load. Peña *et al.*^29^ found that the biaxial response of the arterial wall inferred from constitutive modelling of uniaxial relationships poorly represented the experimental biaxial response of arteries. However, when fitting uniaxial stress–stretch relationships, they assumed the deformation in the other two principal directions (e.g., axial and radial in circumferential uniaxial tests) to be equal, but this is not the case in an isotropic matrix reinforced by fibres located in the circumferential-axial plane of the artery. Furthermore, we used a thin-walled modelling approach, hence, neglecting the bending stiffness and opening angles of the isolated layers. The application of a thick-walled modelling framework would likely improve the accuracy of the estimation of the composite wall mechanical behaviour, but the complexity of the model and data required would also increase considerably.

### Conclusions

In this study, we proposed a novel computational approach where layer-specific mechanical properties, determined experimentally *via* simple and widely available uniaxial testing, are used to formulate a tri-layered model of the arterial wall. When physiological loads were simulated, the model allowed for the in-depth analysis of the contribution of each layer to the overall wall behaviour, highlighting a gradual shift in load bearing from the compliant media to the stiffer adventitia with increasing luminal pressure. In future studies, the application of the proposed modelling framework to human arterial samples could provide valuable insight into the impact of layer-specific remodelling associated with ageing and pathologies on wall mechanics. Further, the limited amount of tissue required in uniaxial tensile tests makes the proposed methodology highly advantageous for human *ex vivo* studies.
